# Serum IgE reduction and paradoxical eosinophilia associated with allergic conjunctivitis after dupilumab therapy

**DOI:** 10.1186/s12348-020-00234-y

**Published:** 2021-02-15

**Authors:** Ayaka Kimura, Ayaka Takeda, Toyo Ikebukuro, Junko Hori

**Affiliations:** 1 Department of Ophthalmology, Nippon Medical School Tama-Nagayama Hospital, 1-7-1, Nagayama, Tama, Tokyo, 206-8512 Japan; 2 Department of Ophthalmology, Nippon Medical School Hospital, 1-1-5, Sendagi, Bunkyo, Tokyo, 113-8603 Japan

## To the Editor,

### Introduction

Dupilumab is a fully human monoclonal antibody against the α subunit of the interleukin (IL)-4 receptor (IL-4Rα), and inhibits IL-4 and IL-13 signalling pathways. These pathways are involved in B-cell differentiation, immunoglobulin (Ig) E production, and a Th-2-dominant immune response [1]. IL-4 and IL-13 are important drivers of various atopic or allergic diseases, such as atopic dermatitis (AD) [2, 3]. Dupilumab has demonstrated efficacy and safety against multiple Th-2-type inflammatory diseases including AD [1–3], asthma [4], chronic rhinosinusitis with nasal polyps [5] and eosinophilic oesophagitis [6] in clinical trials, and has recently been used for AD, asthma, and chronic rhinosinusitis with nasal polyposis when existing treatments fail [7].

Adverse effects of dupilumab have been noted to include pathologies of the ocular surface, including conjunctivitis, blepharitis, keratitis, eye pruritus, dry eye, nasopharyngitis, upper respiratory tract infection, herpes simplex virus, exacerbation of AD, injection-site infection, facial redness, alopecia, and arthralgia [8, 9]. Biologic therapies such as dupilumab inhibit certain cytokines and suppress inflammation of the target organs, but often paradoxically induce or enhance inflammation in other organs. These phenomena are called “paradoxical reactions” [10].

Head and neck erythema have been reported as paradoxical reactions after dupilumab therapy for AD [11]. Histological examination of skin biopsies of such erythema have revealed a psoriasiform reaction pattern suggestive of a drug-induced skin reaction [11]. The frequency of conjunctivitis in clinical trials of dupilumab and in real-world data from a systematic review and meta-analysis have been reported as 8.6–22.1% and 26.1%, respectively [8, 9]. This report presents a case of allergic conjunctivitis associated with eosinophilia as paradoxical reactions induced by dupilumab therapy for AD.

### Case report

**Fig. 1:**
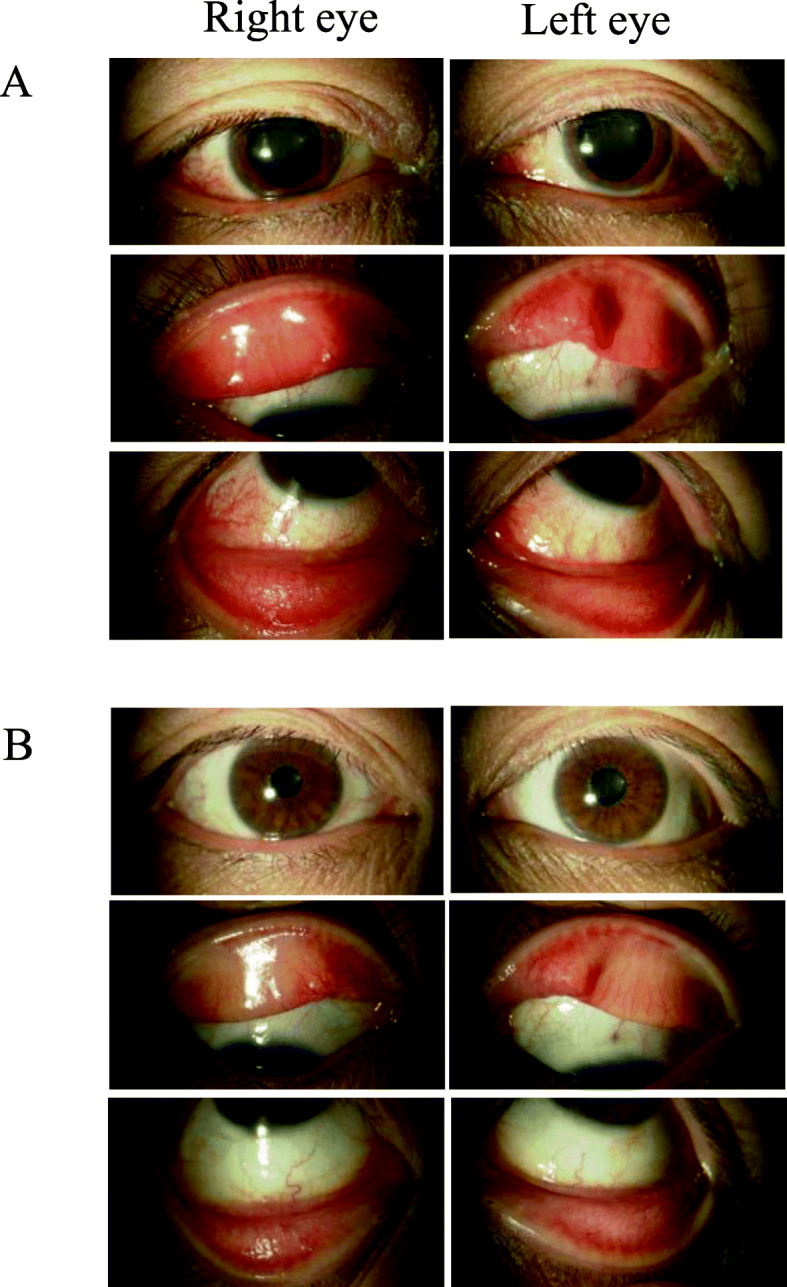
Clinical findings of bilateral blepharitis and conjunctivitis after dupilumab. Bilateral blepharitis, bilateral conjunctival hyperemia, and papillary hyperplasia were observed at 10 weeks after dupilumab administration for AD (**a**). These findings disappeared at 30 weeks by treatment with 0.1% cyclosporine eye drops and 0.1% methylprednisolone ointment (**b**)

A 46-year-old woman was referred to the ocular inflammation service at Nippon Medical School Tama-Nagayama Hospital, for bilateral red eyes and itchiness. She was undergoing a sixth cycle of dupilumab in 10 weeks for AD. Slit lamp examination revealed bilateral conjunctive hyperemia, papillary hyperplasia, and bilateral blepharitis (Fig. 1a). No intraocular inflammation was evident in either eye. She had no past ocular history.

**Fig. 2:**
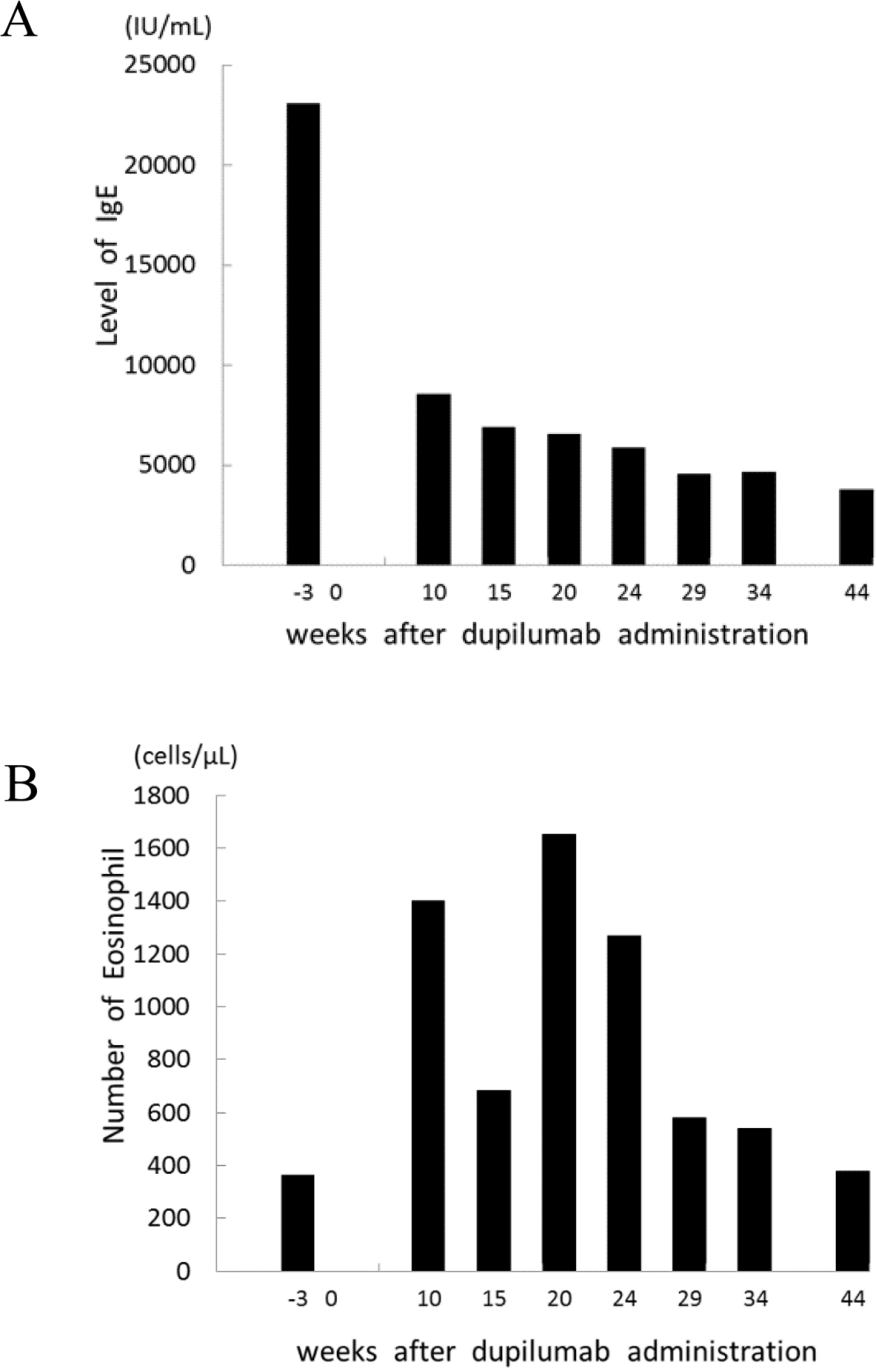
Decreased levels of IgE and paradoxical transient eosinophilia in peripheral blood after dupilumab therapy. IgE level decreased markedly from 23,100 IU/mL at 3 weeks before to 8520 IU/mL at 10 weeks after dupilumab administration (**a**). The number of eosinophils paradoxically increased from 365 cells/μL at 3 weeks before to 1400 cells/μL at 10 weeks after dupilumab administration (**b**)

Blood tests revealed a high eosinophil count (1400 cells/μL) and a high concentration of IgE (8520 IU/mL) at her first visit to our ocular inflammation service. Atopic dermatitis was improved after dupilumab administration, with the Investigator Global Assessment scale for Atopic Dermatitis (IGA) score improved from 4 before dupilumab administration to 2 at 10 weeks after starting dupilumab therapy. However, at the same time as improvement of dermatitis, allergic conjunctivitis paradoxically occurred. When the conjunctivitis appeared (i.e., after 10 weeks of dupilumab administration), serum IgE levels were lower than before dupilumab administration (Fig. 2a). If the allergic conjunctivitis had been a response to pollen or other antigens, serum IgE levels should have been increased, as serum IgE levels in patients with allergic conjunctivitis have been reported as high [12]. In addition, the patient had no nasal symptoms suggestive of allergic rhinitis, such as runny nose or nasal congestion. We therefore ruled out allergic conjunctivitis due to pollen or other antigens.

Allergic conjunctivitis as a paradoxical reaction induced by dupilumab was diagnosed, and she was treated with topical 0.1% cyclosporine eye drops 4 times/day and 0.1% methylprednisolone ointment twice/day for eyelids. Conjunctivitis and blepharitis gradually improved after starting these treatments, and disappeared at 30 weeks (Fig. 1b). She continued to receive dupilumab therapy, which led to decreased levels of serum Th2-type chemokines such as thymus and activation-regulated chemokine (TARC) from 470 to 241 pg/mL by 34 weeks after dupilumab therapy, indicating reduced severity of AD.

It is worth noting that laboratory data showed inverse changes in eosinophils and IgE before and after starting dupilumab (Fig. 2). IgE levels decreased markedly from 23,100 IU/mL at 3 weeks before to 8520 IU/mL at 10 weeks after dupilumab administration (Fig. 2a). On the other hand, eosinophil count paradoxically increased from 365 cells/μL to 1400 cells/μL (Fig. 2b). Conjunctivitis occurred concomitant with eosinophilia.

### Discussion

Dupilumab has been reported to induce eosinophilia with a peak at around 4 weeks after administration [13]. In clinical trials of dupilumab for AD patients, 155 of 465 patients showed eosinophilia at 4 weeks and 3 of 465 patients showed Grade 3 eosinophilia (> 5 × 10^9^ cells/L) [13]. Inhibition of eosinophil recruitment from peripheral blood to inflamed skin tissues by dupilumab has been reported as a mechanism underlying increased eosinophils in peripheral blood [13].

Eosinophils are major pathogenic immune cells in allergic conjunctivitis [14]. Dupilumab-mediated eosinophilia as a paradoxical reaction may be one of the potential mechanisms behind allergic conjunctivitis induced by dupilumab [14]. Further studies are needed to evaluate whether dupilumab-mediated eosinophilia led to eosinophil infiltration into the conjunctiva. Risk factors for the development of dupilumab-associated conjunctivitis have been reported to include AD severity, history of conjunctivitis and elevated levels of biomarkers such as TARC, IgE, and eosinophil counts in peripheral bood [8]. The patient in this report was obviously a high-risk patient, showing high AD severity with an Investigator Global Assessment scale for Atopic Dermatitis score of 4 and elevated levels of both IgE and eosinophils.

### Conclusion

We examined serum IgE, eosinophil counts, and clinical findings of conjunctivitis after dupilumab therapy for more than 6 months in a single patient. This is the first report to describe serum IgE reduction and eosinophilia occurred simultaneously in a patient with allergic conjunctivitis associated with paradoxical eosinophilia after dupilumab therapy. Topical cyclosporine eye drops and steroid ointment offered significant efficacy against dupilumab-associated conjunctivitis, and enabled the AD patient to continue dupilumab therapy.

## Data Availability

Not applicable.

## Abbreviations

IL: Interleukin
IL-4Rα: Interleukin-4 receptor alpha
Ig: Immunoglobulin
AD: Atopic dermatitis
TARC: Thymus and activation-regulated chemokine
